# Genome-Wide Copy Number Variation in Epilepsy: Novel Susceptibility Loci in Idiopathic Generalized and Focal Epilepsies

**DOI:** 10.1371/journal.pgen.1000962

**Published:** 2010-05-20

**Authors:** Heather C. Mefford, Hiltrud Muhle, Philipp Ostertag, Sarah von Spiczak, Karen Buysse, Carl Baker, Andre Franke, Alain Malafosse, Pierre Genton, Pierre Thomas, Christina A. Gurnett, Stefan Schreiber, Alexander G. Bassuk, Michel Guipponi, Ulrich Stephani, Ingo Helbig, Evan E. Eichler

**Affiliations:** 1 Department of Pediatrics, University of Washington, Seattle, Washington, United States of America; 2 Department of Genome Sciences, University of Washington, Seattle, Washington, United States of America; 3 Department of Neuropediatrics, Christian-Albrechts University of Kiel and University Medical Center Schleswig-Holstein, Kiel, Germany; 4 Center for Medical Genetics, Ghent University Hospital, Ghent, Belgium; 5 Institute of Clinical Molecular Biology, Christian-Albrechts University, Kiel, Germany; 6 Department of Genetic Medicine and Development, University of Geneva Medical School and University Hospitals of Geneva, Geneva, Switzerland; 7 Centre Saint Paul-Hôpital Henri Gastaut, Marseilles, France; 8 Unité Fonctionnelle EEG-Epileptologie and Service de Neurologie, Hôpital Pasteur, Nice, France; 9 Department of Neurology, Washington University, St. Louis, Missouri, United States of America; 10 Department of Pediatrics, University of Iowa, Iowa City, Iowa, United States of America; 11 Howard Hughes Medical Institute, University of Washington, Seattle, Washington, United States of America; The Jackson Laboratory, United States of America

## Abstract

Epilepsy is one of the most common neurological disorders in humans with a prevalence of 1% and a lifetime incidence of 3%. Several genes have been identified in rare autosomal dominant and severe sporadic forms of epilepsy, but the genetic cause is unknown in the vast majority of cases. Copy number variants (CNVs) are known to play an important role in the genetic etiology of many neurodevelopmental disorders, including intellectual disability (ID), autism, and schizophrenia. Genome-wide studies of copy number variation in epilepsy have not been performed. We have applied whole-genome oligonucleotide array comparative genomic hybridization to a cohort of 517 individuals with various idiopathic, non-lesional epilepsies. We detected one or more rare genic CNVs in 8.9% of affected individuals that are not present in 2,493 controls; five individuals had two rare CNVs. We identified CNVs in genes previously implicated in other neurodevelopmental disorders, including two deletions in *AUTS2* and one deletion in *CNTNAP2*. Therefore, our findings indicate that rare CNVs are likely to contribute to a broad range of generalized and focal epilepsies. In addition, we find that 2.9% of patients carry deletions at 15q11.2, 15q13.3, or 16p13.11, genomic hotspots previously associated with ID, autism, or schizophrenia. In summary, our findings suggest common etiological factors for seemingly diverse diseases such as ID, autism, schizophrenia, and epilepsy.

## Author Summary

Epilepsy, a common neurological disorder characterized by recurrent seizures, affects up to 3% of the population. In some cases, the epilepsy has a clear cause such as an abnormality in the brain or a head injury. However, in many cases there is no obvious cause. Numerous studies have shown that genetic factors are important in these types of epilepsy, but although several epilepsy genes are known, we can still only identify the genetic cause in a very small fraction of cases. In order to identify new genes that contribute to the genetic causes of epilepsy, we searched the human genome for deletions (missing copies) and duplications (extra copies) of genes in ∼500 patients with epilepsy that are not found in control individuals. Using this approach, we identified several large deletions that are important in at least 3% of epilepsy cases. Furthermore, we found new candidate genes, some of which are also thought to play a role in other related disorders such as autism and intellectual disability. These genes are candidates for further studies in patients with epilepsy.

## Introduction

Epilepsy is one of the most common neurological disorders in humans with a prevalence of ∼1% and a lifetime incidence of up to 3% [1]. The epilepsies present with a broad range of clinical features, and over 50 distinct epilepsy syndromes are now recognized. Particularly in a pediatric setting, a broad range of different epilepsy syndromes can be distinguished. Seizure disorders can roughly be divided into idiopathic or symptomatic epilepsies. While symptomatic epilepsies are due to an identifiable cause such as metabolic disorders, brain trauma or intracranial tumors, idiopathic seizure disorders occur in the absence of identifiable causal factors and are thought to have a strong genetic contribution.

Although it has long been observed that the idiopathic epilepsies have a genetic component, the genetic etiology of only a small fraction of cases can be determined. The role of copy number variants (CNVs) in intellectual disability (ID) [2]–[8], autism [9]–[14] and schizophrenia [15]–[19] has been extensively investigated. It has become increasingly clear that, collectively, rare variants contribute significantly to the etiology of these common diseases–following the rare variant common disease hypothesis. We hypothesize this can be extended to other neurological disorders and that rare CNVs significantly contribute to the genetic etiology of epilepsy.

Recently, in a study targeted to six genomic regions, recurrent microdeletions on chromosome 15q13.3, 16p13.11 and 15q11.2 were identified as important genetic factors predisposing to idiopathic generalized epilepsy (IGE) [20]–[22]. Here, we carry out whole-genome array comparative genomic hybridization (CGH) in a cohort of 517 individuals with mixed types of idiopathic epilepsy in order to discover novel copy number changes associated with epilepsy. We find recurrent microdeletions of 15q13.3, 16p13.11 and 15q11.2 each in ∼1% of affected individuals, confirming previous studies [20]–[22]. In addition to recurrent rearrangements at rearrangement-prone regions, we show that, overall, 8.9% of affected individuals have one or more rare copy number changes involving at least one gene.

## Results

We performed genome-wide array CGH to detect copy number changes in 517 patients with mixed types of epilepsy. Of these, 399 have idiopathic generalized epilepsy (IGE), 50 have benign epilepsy with centrotemporal spikes (BECTS) and 68 have other types of idiopathic seizure disorders (Table 1). We used a custom microarray with high-density targeted coverage of 107 regions of the genome flanked by large, highly homologous duplications, termed rearrangement hotspots [23]. In addition, probes were evenly spaced throughout the remainder of the genome with average probe spacing of ∼38 kb. Overall, we find that 46 probands (8.9%) carry one or more rare CNVs not previously reported in the 2493 unrelated controls [24]. The rare CNVs detected in our cohort range in size from 13 kb to 15.9 Mb (average 1.2 Mb; median 600 kb), and the majority (69%) are deletion events.

**Table 1:** Phenotypes of probands evaluated by array CGH.

Type of epilepsy	N	Hotspot CNVs detected	Other CNVs detected	Total
**IGE (n = 399)**				
Juvenile myoclonic epilepsy (JME)	189	8∧	9∧	17
Absence epilepsy (AE)	94	5	5*	10
IGE with GTCS only	33	0	2	2
IGE unclassified	63	2	4	6
Benign myoclonic epilepsy of infancy	5	0	0	0
Myoclonic astatic epilepsy (MAE)	15	0	2	2
**Idiopathic focal epilepsy (n = 63)**				
BECTS	50	3	2	5
ABPE	13	0	0	0
**Other (n = 55)**				
ESES	4	0	0	0
Landau-Kleffner syndrome	3	0	0	0
Severe IGE of infancy (SIGEI)	15	1	1	2
West syndrome	4	0	2*	2
IC/NC	10	1	2*	3
Unclassified	19	0	2	2
**Total**	**517**	**20**	**31**	**51**

IGE, idiopathic generalized epilepsy; GTCS, generalized tonic-clonic seizures; BECTS, benign epilepsy with centrotemporal spikes; ABPE, atypical benign partial epilepsy; ESES, electrical status epilepticus during slow-wave sleep; IC, infantile convulsions; NC, neonatal convulsions;

*indicates two events in a single individual;

**∧:** two individuals (EMJ071 and EMJ117) each carrying one hotspot and one non-hotspot event.

### Rearrangements at genomic hotspots

**Figure 1:**
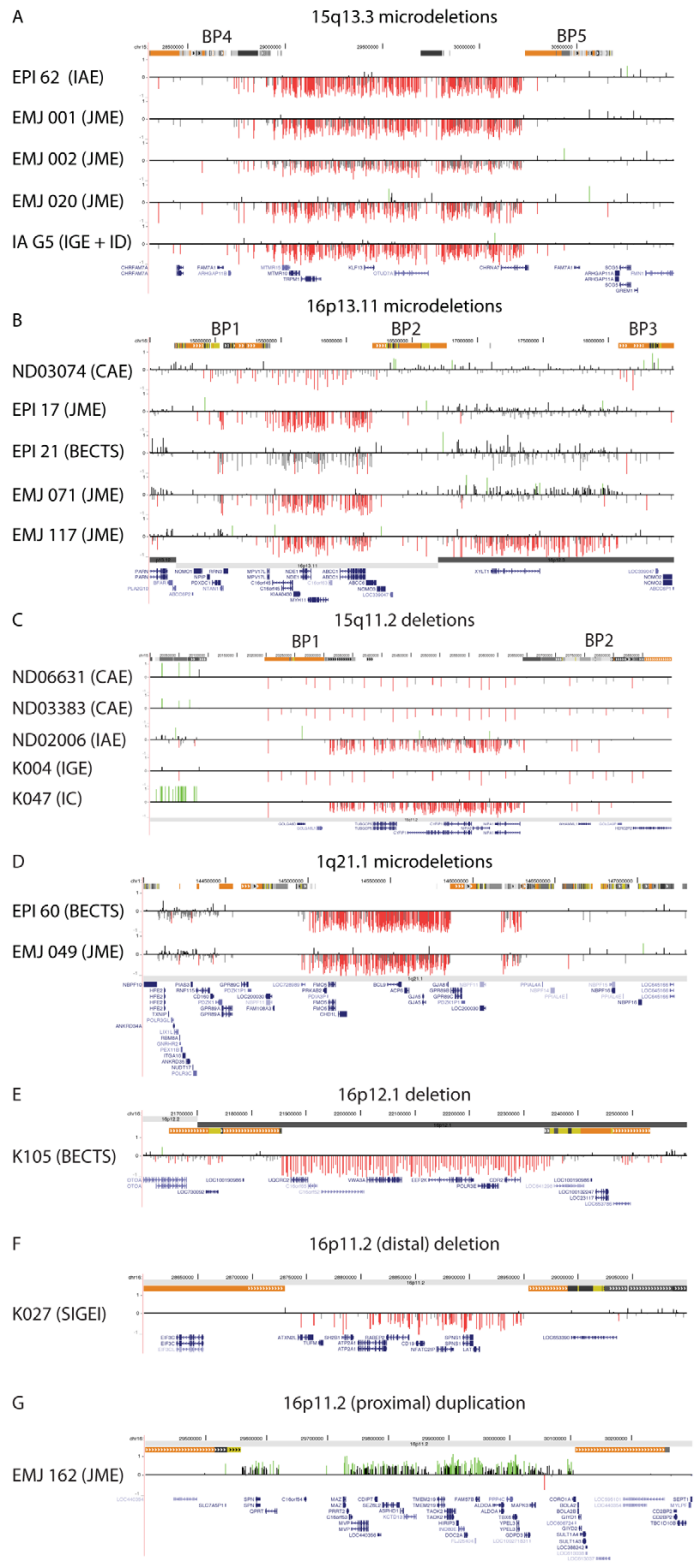
Deletions and duplications at genomic rearrangement hotspots in 20 probands. Array CGH results are depicted for (A) 15q13.3, chr15: 28.0–31.0 Mb, (B) 16p13.11, chr16: 14.5–18.5 Mb, (C) 15q11.2, chr15: 20.0–20.9 Mb, (D) 1q21.1, chr1: 144.0–147.5 Mb, (E) 16p12.1, chr16: 21.6–22.6 Mb, (F) 16p11.2, chr16:28.6–29.1 Mb, and (G) 16p11.2, chr16: 29.0–30.3 Mb. For each individual, deviations of probe log_2_ ratios from 0 are depicted by gray and black lines. Those exceeding a threshold of 1.5 s.d. from the mean probe ratio are colored green and red to represent relative gains and losses, respectively. Segmental duplications of increasing similarity (90–98%, 98–99%, and >99%) are represented by gray, yellow, and orange bars, respectively. RefSeq genes are depicted in blue.

We first evaluated rearrangement hotspots for copy number changes. We found 20 probands (3.9%) with copy number changes at known rearrangement hotspots including 15q13.3 deletions (n = 5), 16p13.11 deletions (n = 5), 15q11.2 BP1–BP2 deletions (n = 5), 1q21.1 deletions (n = 2), a 16p12.1 deletion (n = 1), a 16p11.2 duplication (n = 1) and a more distal 16p11.2 deletion (n = 1) (Table 2, Figure 1). We also identified four individuals with duplications of 15q11.2 BP1–BP2; because duplications of this region are frequent in the general population, we classified these duplications as polymorphic events. These results confirm our previous studies and emphasize the importance of deletions of 15q13.3, 16p13.11 and 15q11.2 BP1–BP2 as frequent genetic susceptibility factors in epilepsy [20]–[22]. All three regions have also been associated with ID, autism and/or schizophrenia [15], [17], [25]–[32], as have deletions at 1q21.1 [33], [34], two distinct regions of 16p11.2 [10], [14], [35]–[37] and 16p12 [38], which were also detected in our cohort. Deletions of 16p13.11 (5/517 vs 0/2493 controls, p = 0.00014, Fisher's exact test), 15q13.3 (5/517 vs 0/2493, p = 0.00014) and 15q11.2 (5/517 vs. 4/2493, p = 0.010) are significantly enriched in our epilepsy cohort and together account for 2.9% of cases.

**Table 2:** Rare copy number variants in 517 patients with epilepsy.

Case	Chromosome Location	HS	Coordinates (build36; Mb)	Size	CNV	Inheritance	Phenotype	*RefSeq* genes (n)	Possible candidate genes
**Idopathic Generalized Epilepsies (n = 399)**									
EMJ 049	1q21.1	Y	Chr1: 145.0–145.9	900 kb	Del	-	JME	8	*GJA8*
ND02006	15q11.2	Y	Chr15: 20.2–20.8	600 kb	Del	-	IAE	4	*CYFIP1*
ND03383	15q11.2	Y	Chr15: 20.2–20.8	600 kb	Del	-	CAE	4	*CYFIP1*
ND06631	15q11.2	Y	Chr15: 20.2–20.8	600 kb	Del	Inh (P)	CAE	4	*CYFIP1*
K004	15q11.2	Y	Chr15: 20.2–20.8	600 kb	Del	Inh (P)	IGE	4	*CYFIP1*
EPI 62	15q13.3	Y	Chr15: 28.7–30.1	1.4 Mb	Del	-	IAE	6	*CHRNA7*
EMJ 001**	15q13.3	Y	Chr15: 28.7–30.1	1.4 Mb	Del	-	JME	6	*CHRNA7*
EMJ 002	15q13.3	Y	Chr15: 28.7–30.1	1.4 Mb	Del	-	JME	6	*CHRNA7*
EMJ 020**	15q13.3	Y	Chr15: 28.7–30.1	1.4 Mb	Del	-	JME	6	*CHRNA7*
IA G5	15q13.3	Y	Chr15: 28.7–30.1	1.4 Mb	Del	-	IGE + ID	6	*CHRNA7*
EMJ 162	16p11.2	Y	Chr16: 29.5–30.2	700 kb	Dup	-	JME	30	*SEZ6L2*
ND3074	16p13.11	Y	Chr16:15.4–16.3	900 kb	Del	Inh (M)	CAE	6	*NDE1*
EPI 17	16p13.11	Y	Chr16:15.4–16.3	900 kb	Del	-	JME	6	*NDE1*
EMJ 071*	16p13.11	Y	Chr16:15.4–16.3	900 kb	Del	-	JME	6	*NDE1*
EMJ 117*	16p13.11	Y	Chr16: 15.4–18.5	3.1 Mb	Del	-	JME	7	*NDE1*
ND05586	1p31.1		Chr1: 72.04–72.15	111.3 kb	Del	Inh (P)	CAE	1	*NEGR1*
ND05260*	4q22.2		Chr4: 94.18–94.83	646.6 kb	Del	Inh (M)	CAE	1	*GRID2*
K 111	5p15.33		Chr5: 0.72–1.43	713.0 kb	Dup	Inh (M∧)	MAE	10	*NKD2, SLCA18*
EP007.1	5q33.2		Chr5: 153.2–160.3	7.1 Mb	Del	Not in M	IGE + ID	44	*CYFIP2*
EMJ 005	6q12		Chr6: 65.03–66.09	1.06 Mb	Dup	-	JME	1	*EYS*
ND01440	7q11.22		Chr7: 69.38–69.46	78.7 kb	Del	-	JME	1	*AUTS2*
K 039	7q36.1		Chr7: 151.35–151.43	85.8 kb	Del	Inh (P)	MAE	1	*GALNT11*
ND03578	8q21-q22		Chr8: 83.97–97.20	15.9 Mb	Dup	Inh (P)	JME+ID	50	*many*
EMJ 013	9p21.3		Chr9: 21.21–21.63	427.5 kb	Del	-	JME	9	*KLHL9*
EP005.1	9q21.32		Chr9: 83.9–85.2	1.30 Mb	Del	Inh (M)	IGE	2	*RASEF*
ND05260*	9q31.3		Chr9: 113.33–114.33	1.01 Mb	Dup	Inh (M)	CAE	10	
EMJ 071*	13q31.1		Chr13: 84.69–85.36	671.8 kb	Del	-	JME	1	*SLITRK6*
EMJ 067	14q24.2		Chr14: 70.96–71.23	268.6 kb	Del	-	JME	1	*SIPA1L1*
EPI 66	15q25.2		Chr15: 83.00–83.12	117.4 kb	Dup	-	IAE	3	*NBM*
ND03244	16q23.1		Chr16: 74.49–75.27	785.8 kb	Dup	-	GTCS only	1	*CNTNAP4*
EPI 52	17p11.2		Chr17: 19.92–19.94	13.3 kb	Del	-	GTCS only	1	*CYTSB*
EMJ 117*	17p11.2		Chr17: 19.92–19.94	17.5 kb	Del	-	JME	1	*CYTSB*
EPI 40	17q12		Chr17: 30.53–30.87	338.5 kb	Del	-	IAE	7	*UNC45*
EMJ 039	18q11.2		Chr18: 19.66–20.50	840.4 kb	Dup	-	JME	6	
EMJ 069	18q11.2		Chr18: 19.66–20.50	840.4 kb	Dup	-	JME	6	
ND02416	21q21.1		Chr21: 16.21–18.81	2.59 Mb	Dup	Inh (M)	IGE + ID	7	
EPI 26	Xp22.31		ChrX: 7.78–8.39	605.5 kb	Dup	-	IGE	4	*PNPLA4*
**Idiopathic Focal Epilepsies (n = 63)**									
EPI 60	1q21.1	Y	Chr1: 145.0–145.9	900 kb	Del	-	BECTS	8	
K 105	16p12.1	Y	Chr16: 21.8–22.3	500 kb	Del	-	BECTS	7	
EPI 21	16p13.11	Y	Chr16: 15.4–16.3	900 kb	Del	-	BECTS	6	*NDE1*
EPI 58	4q35.1		Chr4: 186.30–186.61	302.4 kb	Dup	-	BECTS	8	*SLC25A, SNX25*
K 093	8p23.1		Chr8: 10.19–10.37	173.1 kb	Del	-	BECTS	1	*MSRA*
**Other (n = 55)**									
K 047	15q11.2	Y	Chr15: 20.2–20.8	600 kb	Del	brother∧	IC	4	*CYFIP1*
K 027	16p11.2	Y	Chr16:28.7–28.9	200 kb	Del	-	SIGEI	9	
K 109	2q35		Chr2: 218.36–218.94	571.9 kb	Dup	-	SIGEI	11	
EPI 51*	5q35.1		Chr5: 167.62–167.89	268.7 kb	Dup	-	West	4	*WWC1*
EPI 51*	5q35.1		Chr5: 169.43–169.64	230.0 kb	Dup	-	West	4	*DOCK2, FOXI1*
K 054	7q11.22		Chr7: 69.38–69.42	38.3 kb	Del	-	Unclassified	1	*AUTS2*
K034*	7q35		Chr7:146.06–146.36	304.4 kb	Del	Inh (P∧)	NC	1	*CNTNAP2*
ND08273	15q13.3-q14		Chr15: 30.66–32.44	1.78 Mb	Dup	Inh (M)	Unclassified	15	
K034*	17p13.1		Chr17:10.36–10.72	370 kb	Del	Inh (P∧)	NC	7	*MYH1-3; SCO*

HS, hotspot region; Del, deletion; Dup, duplication; Inh, inherited; M, maternal; P, paternal;

**∧:** affected; -, parents unavailable; JME, juvenile myoclonic epilepsy; IAE, idiopathic absence epilepsy; CAE childhood absence epilepsy; IGE, idiopathic generalized epilepsy; GTCS, generalized tonic clonic seizures only; ID, intellectual disability; BECTS, benign epilepsy with centrotemporal spikes; IC, infantile convulsions; SIGEI, several idiopathic generalized epilepsy of infancy; NC, neonatal convulsions;

*two CNVs detected in same individual;

**15q13 deletions previously detected by MLPA [60].

### Rare or unique deletions involving potential candidate genes

We next focused on non-hotspot CNVs that overlap one or more genes and are not present in the control cohort of 2493 individuals [24]. We identified 28 individuals with at least one rare gene-containing deletion or duplication, and five individuals each carry two rare CNVs (Table 2). Fifteen of the events we detected involve a single gene. Two genes were altered in two patients each: *AUTS2* deletions were identified in one proband with juvenile myoclonic epilepsy (JME) and one proband with unclassified non-lesional epilepsy with features of atypical benign partial epilepsy (ABPE) [39]. Deletions involving *CTYSB* (*SPECC1*) were identified in two probands with IGE. All other single-gene CNVs were seen only once. Seventeen events involved multiple genes, one of which was observed in two different individuals with JME (duplication of 18q11, Table 2).

### Individuals with multiple rare CNVs

**Figure 2:**
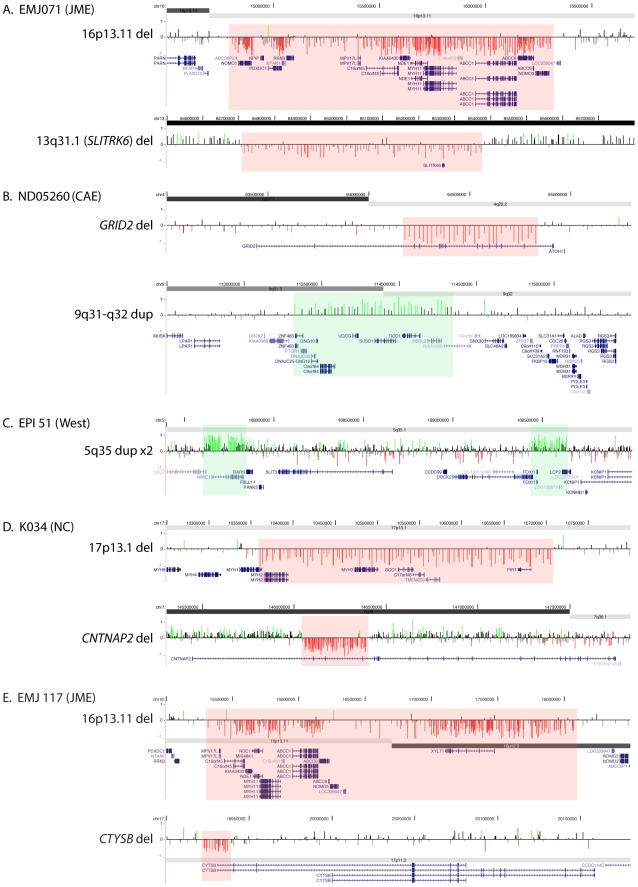
Two rare CNVs in five probands. Array CGH results are shown for the for two rare CNVs detected in probands EMJ071 (A), ND05260 (B), EPI51 (C), K034 (D), and EMJ117 (E). Array CGH results are depicted as in Figure 1; segmental duplications are not shown in this figure.

We found five individuals with two rare CNVs (Figure 2). Two patients with JME and a deletion of 16p13.11 (EMJ071 and EMJ117) each have a second rare deletion. EMJ071 has a large deletion on chromosome 13 that removes the *SLITRK6* gene, a member of the SLITRK gene family involved in controlling neurite outgrowth; individual EMJ117 also has a deletion involving the *CTYSB* gene. Case ND05260 (childhood absence epilepsy, CAE) carries a 647-kb deletion within the *GRID2* gene, which encodes a glutamate receptor expressed in the cerebellum, and a 1-Mb duplication of 9q31. Though both are maternally inherited, neither has been reported in controls. Case EPI 51 (idiopathic West syndrome) has two apparently independent duplications of chromosome 5q35, each containing several genes. Finally, we identified one proband with neonatal convulsions (NC) carrying a deletion within the *CNTNAP2* gene that spans exons 2–4 as well as a 370-kb deletion of 17p13 involving 7 genes.

DNA from one of more family members was available for analysis in 14 cases. Inheritance, if determined, is shown in Table 2. In twelve cases, we determined that one or both CNVs in the proband were inherited; in three cases the transmitting parent is also affected. In one case (EP007.1), the CNV was not found in the mother, but the father was unavailable. In another case (K047), parents were unavailable, but a brother was found to carry the same CNV suggesting one of the parents carries the same CNV.

## Discussion

In this study, we performed whole-genome array CGH in a series of 517 individuals with a presenting diagnosis of idiopathic epilepsy in order to discover novel copy number changes associated with epilepsy. While our previous studies were targeted to specific genomic regions in probands with IGE [21], [22], here we present data from whole-genome analysis on probands with IGE and extend our analysis to other idiopathic epilepsy syndromes. In total, we identified 46 individuals (8.9%) with 51 rearrangements that may be pathogenic as they were not found in controls or were significantly enriched in our epilepsy cohort.

### Hotspot rearrangements

Rearrangements at several genomic hotspots have been associated with a range of neurocognitive disorders. In our cohort of 517 probands with epilepsy, we find deletions at 15q13.3, 16p13.11 and 15q11.2 in 2.9% of our cases. Interestingly, all of the deletions of 15q13.3 (n = 5) and 4/5 deletions at 16p13.11 and 15q11.2 were in probands with IGE, accounting for 3.3% of the patients with IGE in our cohort confirming our previous findings. While it is possible that deletions of 15q13.3 are also predisposing to non-IGE epilepsy syndromes, we did not find this to be the case in our series (n = 118). Additional large cohorts of patients with focal epilepsy or epileptic encephalopathy will be required to determine whether these deletions also play a significant role in other subtypes of epilepsy.

Deletions of 16p13.11 have previously been associated with intellectual disability +/− congenital anomalies in one study [26]. Three of four probands with 16p13.11 deletions in that series had epilepsy; two further fetal cases had brain abnormalities. The findings in this cohort and one previous study of IGE [20] suggest that deletions of 16p13.11 are more frequent in epilepsy (0.5–1% of cases) than in other phenotypes including ID and autism [26], [27], [32], and may be as frequent as 15q13.3 deletions in individuals with IGE. Deletions and duplications of this region have also been reported in schizophrenia, though the associations have not been statistically significant [16], [29].

Deletions of 15q13.3, detected in five individuals with IGE in our series, have been associated with a wide range of phenotypes including ID, autism, epilepsy and schizophrenia [15], [17], [20]–[22], [25], [28], [30], [31], [40]. The gene within the 15q13.3 region that is most likely responsible for the epilepsy phenotype is *CHRNA7*, a subunit of the nicotinic acetylcholine receptor. At least two small studies have failed to identify causal point mutations in the *CHRNA7* gene in autosomal dominant nocturnal frontal lobe epilepsy [41] and JME [42], but additional studies should be performed to further evaluate affected individuals for mutations. A recent publication identifying atypical rearrangements with exclusive deletions of *CHRNA7* further emphasizes the importance of *CHRNA7* as the main candidate gene in this region [43].

Compared to the above structural genomic variants, copy number variation at 15q11.2 between breakpoints BP1 and BP2 of the Prader-Willi and Angelman syndrome region is more common in the general population with the BP1–BP2 deletion present in 0.2% of unaffected individuals. Despite this, deletions between BP1 and BP2 have now been reported as enriched in patients with schizophrenia [16], [17], ID [27] and epilepsy [20]. Furthermore, there is evidence that patients with Prader-Willi or Angelman syndrome who have deletions including BP1–BP2 are more severely affected [44]–[46]. In this study, we also find enrichment of deletions at this locus in affected individuals. Together, these studies suggest that deletion of the 15q11.2 BP1–BP2 region confers susceptibility to a wide range of neuropsychiatric conditions, albeit with incomplete penetrance.

Two patients in our series, one each with JME and BECTS, have deletions of 1q21.1, which have been previously associated with a wide range of phenotypes, including intellectually disability and developmental delay [33], [34], schizophrenia [15], [17], [18], congenital heart disease [47], [48] and cataracts [34], [49]. In two large studies of patients who present primarily with cognitive or developmental delay, 5/42 (11.9%) patients also had seizures [33], [34]; 1 of 10 patients with schizophrenia and a 1q21.1 deletion also had epilepsy [15]. Identifying 1q21.1 microdeletions in patients with idiopathic generalized and idiopathic focal epilepsies suggests that variation at this locus predisposes to a broad range of seizure disorders crossing traditional diagnostic boundaries.

In addition, we identified one patient (EMJ162) with JME and a duplication of 16p11.2 (chr16: 29.5–30.2 Mb), which has been associated with autism, developmental delay and schizophrenia [10]–[12], [14], [27], [35], [37]. Finally, we identified one individual with severe idiopathic generalized epilepsy of infancy (SIGEI) (K027) with a more distal deletion of 16p11.2 (chr16: 27.7–28.9 Mb), recently associated with severe early-onset obesity and ID [36], and one patient with BECTS (K105) and a deletion of 16p12.1 (chr16: 20.2–20.8 Mb), also associated with ID and other neurodevelopmental defects [38]. Thus, our data adds to the phenotypic spectrum associated with rearrangements at several genomic hotspot regions. In particular, we identify hotspot deletions in two patients with BECTS. Gene identification in BECTS, despite representing the most common focal epilepsy syndrome of childhood, has been elusive so far. Here, we suggest that some recurrent hotspot deletions might predispose to both idiopathic generalized and focal epilepsies.

### Non-hotspot rearrangements

We detected 18 deletions and 16 duplications that are not associated with rearrangement hotspots. Fifteen events involve a single gene; of these, 12 are deletions. Although all of the CNVs reported here are not found in our control set of 2493 individuals, it is possible that some are rare but benign CNVs. However, many of the CNVs we identified contain one of more plausible candidate genes for epilepsy (Table 2).

We identified a deletion of exons 2–4 in the *CNTNAP2* gene in a proband with neonatal seizures. *CNTNAP2* has been identified as a candidate gene for autism [50]–[52], and heterozygous deletions involving the gene were reported in three patients with schizophrenia and autism [53]. The deletion is predicted to cause an in-frame deletion of 153 amino acids in the resulting protein. The same patient has a 370-kb deletion of 17p13 that deletes seven genes and has not been seen in our control cohort. We also identified a patient with a duplication encompassing a related gene, *CNTNAP4*. Finally, two individuals in our cohort have overlapping deletions within *AUTS2*. This gene is disrupted by *de novo* balanced translocations in three unrelated individuals with mental retardation [54] and a pair of twins with autism and mental retardation [55], suggesting a role for *AUTS2* in normal cognitive development. The two deletions we detected are intragenic and overlapping.

### CNVs in epilepsy subtypes

Previous studies of CNVs in epilepsy have focused on probands with IGE. It is known from studies of families with autosomal dominant epilepsy that a wide range of seizure types can be caused by the same single-gene mutation. For example, Dravet syndrome, a severe early-onset disorder associated with poor cognitive outcome, and the milder generalized epilepsy with febrile seizures plus (GEFS+) syndrome are both caused by mutations in the *SCN1A* gene [56]–[58]. Therefore, we included probands with common idiopathic focal epilepsies and non-lesional, idiopathic epilepsies. Some of our probands were diagnosed with specific epilepsy syndromes, including myoclonic astatic epilepsy (Doose Syndrome), atypical benign partial epilepsy [39], Landau-Kleffner syndrome, idiopathic West syndrome, severe idiopathic generalized epilepsy of infancy [59] and benign neonatal or infantile seizures. These particular epilepsy syndromes are usually associated with normal MRI results. We find that 6.6% of probands with IGE and 7.9% of those with idiopathic focal epilepsy harbor rare CNVs that may underlie their epilepsy phenotype. Notably, 12.7% of patients with other, often more severe forms of epilepsy in our series carry one or more rare CNVs. In our series, the vast majority of patients with deletions of 15q13.3, 16p13.11 and 15q11.2 BP1–BP2 were in the IGE cohort, accounting for 3.3% of cases. In the non-IGE patients, a deletion of 15q11.2 was found in a single patient with infantile seizures and a deletion of 16p13.11 was found in one patient with BECTS, suggesting that deletions at these three genomic hotspots confer greater risk for IGE than other types of epilepsy.

In summary, we find that 46/517 probands (8.9%) with various forms of idiopathic epilepsy carry one or more rare CNVs that may predispose to seizures, a frequency similar to that in studies of patients who present with other neurocognitive phenotypes, including ID, autism and schizophrenia. Furthermore, we identified CNVs involving genes and genomic regions previously identified in patients with the neurocognitive phenotypes listed above, suggesting common genetic etiological factors for these disorders. Our data suggest that rare CNVs are important in many subtypes of idiopathic epilepsies, including idiopathic generalized and idiopathic focal epilepsies as well as specific idiopathic, non-lesional epilepsy syndromes. The genomic regions and genes identified in this study are potential novel candidate genes for epilepsy.

## Materials and Methods

### Ethics statement

Patients were collected at five centers after appropriate human subjects approval and informed consent at each site.

### Patient cohorts

Patients were collected at five centers: (1) 140 probands with a primary diagnosis of JME, CAE, absence epilepsy, IGE or idiopathic epilepsy were selected from the NINDS repository (http://ccr.coriell.org/ninds); (2) 160 patients are probands with a primary diagnosis of JME from Switzerland. Patients from cohorts (1) and (2) were previously analyzed using MLPA for the *CHRNA7* gene [60], and two probands (EMJ001 and EMJ020) were determined to have 15q13.3 microdeletions by that method; they were not previously analyzed for any other copy number changes. (3) 186 German patients came from two cohorts: 76 patients from a population-based cohort from Northern Germany (POPGEN cohort) and 110 patients with childhood-onset epilepsy collected at the University of Kiel. Finally, 41 patients with various idiopathic generalized epilepsies collected at (4) the University of Iowa and (5) at Washington University, St. Louis. DNA from the NINDS repository was derived from cell lines; DNA from all other cohorts was directly from blood. Patients were diagnosed according to the widely used 1989 ILAE classification [61]. In addition, several pediatric patients were diagnosed with specific syndromes not yet recognized in the ILAE classification (Table 1). Patients with non-lesional, idiopathic epilepsies in which diagnostic criteria of the recent ILAE classification for particular epilepsy syndromes were not met were labeled as “unclassified”.

### Array comparative genomic hybridization (CGH)

Array CGH was performed using either custom or commercially available oligonucleotide arrays containing 135,000 isothermal probes (Roche NimbleGen, Inc.). Customized arrays (459 samples) were designed with higher density probe coverage in known rearrangement hotspot regions (average probe spacing 2.5 kb) with lower density whole-genome backbone coverage (average probe spacing 38 kb). A subset of samples (n = 62) was analyzed using a commercially available whole-genome array (Roche NimbleGen 12×135 k whole-genome tiling array) with average probe spacing throughout the genome of 21 kb.

### Data analysis

Data were analyzed according to manufacturer's instructions using NimbleScan software to generate normalized log_2_ fluorescence intensity ratios. Then, for each sample, normalized log intensity ratios are transformed into z-scores using the chromosome-specific mean and standard deviation. Z-scores are subsequently used to classify probes as “increased”, “normal” and “decreased” copy-number using a three-state Hidden Markov Model (HMM). The HMM was implemented using HMMSeg [62], which assumes Gaussian emission probabilities. The “increased” and “decreased” states are defined to have the same standard deviation as the “normal” state but with mean z-score two standard deviations above and below the mean, respectively. Probe-by-probe HMM state assignments are merged into segments according to the following criteria: consecutive probes of the same state less than 50 kb apart are merged, and if two segments of the same state are separated by an intervening sequence of ≤5 probes and ≤10 kb, both segments and intervening sequence are called as a single variant. CNV calls are filtered to eliminate (i) events containing <5 probes, (ii) CNVs with >50% overlap in a series of 2493 control individuals [24] and (iii) events that had no overlap with RefSeq genes. In addition, when comparing CNV calls to control CNVs, we eliminated calls for which there was insufficient probe coverage (<5 probes) in the control data to identify the same or similar CNV. Filtered copy number changes are also visually inspected in a genome browser.
